# Discovering chronic pain treatments: better animal models might help us get there

**DOI:** 10.1172/JCI167814

**Published:** 2023-03-01

**Authors:** Norman E. Taylor, Luiz Ferrari

**Affiliations:** Department of Anesthesiology, The University of Utah, Salt Lake City, Utah, USA.

## Abstract

Only three classes of pain medications have made it into clinical use in the past 60 years despite intensive efforts and the need for nonaddictive pain treatments. One reason for the failure involves the use of animal models that lack mechanistic similarity to human pain conditions, with endpoint measurements that may not reflect the human pain experience. In this issue of the *JCI*, Ding, Fischer, and co-authors developed the foramen lacerum impingement of trigeminal nerve root (FLIT) model of human trigeminal neuralgia that has improved face, construct, and predictive validities over those of current models. They used the FLIT model to investigate the role that abnormal, hypersynchronous cortical activity contributed to a neuropathic pain state. Unrestrained, synchronous glutamatergic activity in the primary somatosensory cortex upper lip and jaw (S1ULp–S1J) region of the somatosensory cortex drove pain phenotypes. The model establishes a powerful tool to continue investigating the interaction between the peripheral and central nervous systems that leads to chronic pain.

## Chronic pain is a serious public health problem

Chronic pain has a profound cumulative impact on our nation, with an estimated 100 million Americans being affected each day, at a cost of over $500 billion in health care and lost productivity each year (1, 2). Treatment of chronic pain remains a challenge because of a lack of clinical biomarkers for the evaluation and management of the patient in pain (3, 4) and a dearth of efficacious therapeutic strategies (5). An increased emphasis on evaluating and managing pain resulted from the Joint Commission’s 2000 recommendation that pain be assessed in all patients. This shift led to an increase in opioid prescriptions, doubling the number of Americans using opioids (1, 5), but did not improve patient-reported pain scores. Instead, it induced an epidemic of opioid dependence and addiction and produced a surge in opioid-related drug overdose deaths (6). This consequence and other deleterious opioid-related effects highlight the critical need for therapies that relieve pain while avoiding harmful side effects.

## Six decades in search of better options

Intensive scientific efforts have focused on discovering and developing such therapies. Nevertheless, there have been very few successes, with only three classes of pain medications being approved by the FDA since placebo-controlled trials were mandated by the United States Congress in 1962 (7). The triptans are the greatest success story, receiving FDA approval for the treatment of migraine headaches in 1992 (8). The other two successes have made a much more modest impact. TRPV1 agonists such as capsaicin are used as topical treatments for minor pain, while ziconotide is used for severe refractory pain. However, the clinical utility of ziconotide is limited by the need for continuous intrathecal infusion and its low therapeutic ratio (9). Local anesthetics, opioids, NSAIDs, corticosteroids, antidepressants, and anticonvulsants were all in use prior to 1962 but continue to be limited by their short duration of action, inadequate efficacy, and/or poorly tolerated adverse events (7).

There has been a robust, ongoing national discussion regarding the cause of the lack of success in translating compounds that appear to be effective in animal models but fail to produce clinically efficacious treatments in people (10, 11). Some of the reasons proposed include (a) a lack of clinically relevant pain models that reflect the genetic, environmental, sex-dependent, and psychologic aspects of human pain conditions (12, 13); (b) a failure in the design of clinical trials due to a lack of biomarker-targeted patient selection, including the identification of placebo responders (14); and (c) the possibility that pain is not unique in the context of other neurological disorders for which translation has been universally poor.

## The development of better rodent pain models

Traditionally, modeling pain conditions in animals — usually rodents — involves the use of chemical or surgical interventions or repeated exposure to an environmental stressor (12, 13). Researchers typically perform such interventions in rodent strains such as the Sprague Dawley rat or the C57BL/6 mouse, which do not have a genetic susceptibility to pain. This practice limits the applicability of findings to humans, as many pain syndromes arise spontaneously without apparent precipitating factors. In response to the national discussion, there have been several rodent models developed recently with an eye to improving face validity (defined as the similarity in clinical signs and symptoms), construct validity (defined as similarity in pathophysiologic disease mechanisms), and predictive validity (defined as the relative effectiveness of various clinical interventions being mimicked by the model). Among these systems, models of complex regional pain syndrome (CPRS) (15), complex orthopedic trauma (16), temporomandibular joint pain (17), and a genetic model of widespread hyperalgesia (18) have been used effectively to determine pathophysiological mechanisms associated with these conditions.

In this issue of the *JCI*, Ding, Fischer, and co-authors (19) describe a different trigeminal neuropathic pain model that exhibits several key features that have been on investigators’ wish lists for years, including (a) face validity: the foramen lacerum impingement of trigeminal nerve root (FLIT) model exhibits robust spontaneous pain behaviors (facial grimacing, excessive facial grooming, intermittent eye squinting) and clinically relevant functional consequences of persistent pain (body weight loss, decreased wood chewing, soft food preference, increased incisor length, increased anxiety behaviors, and sexual dysfunction), which are consistent with the clinical signs and symptoms of human trigeminal neuralgia (TN); (b) construct validity: the FLIT model utilizes a clever, reversible trigeminal nerve root compression that mimics the human pathology of trigeminal nerve root impingement at its entry zone (20); and (c) predictive validity, which includes nearly complete ineffectiveness of NSAIDS, partial effectiveness of carbamazepine, and robust effectiveness of trigeminal nerve root decompression surgery, a definitive treatment for patients with TN with vascular compression (21).

## Interactions of the peripheral and central nervous systems

**Figure 1:**
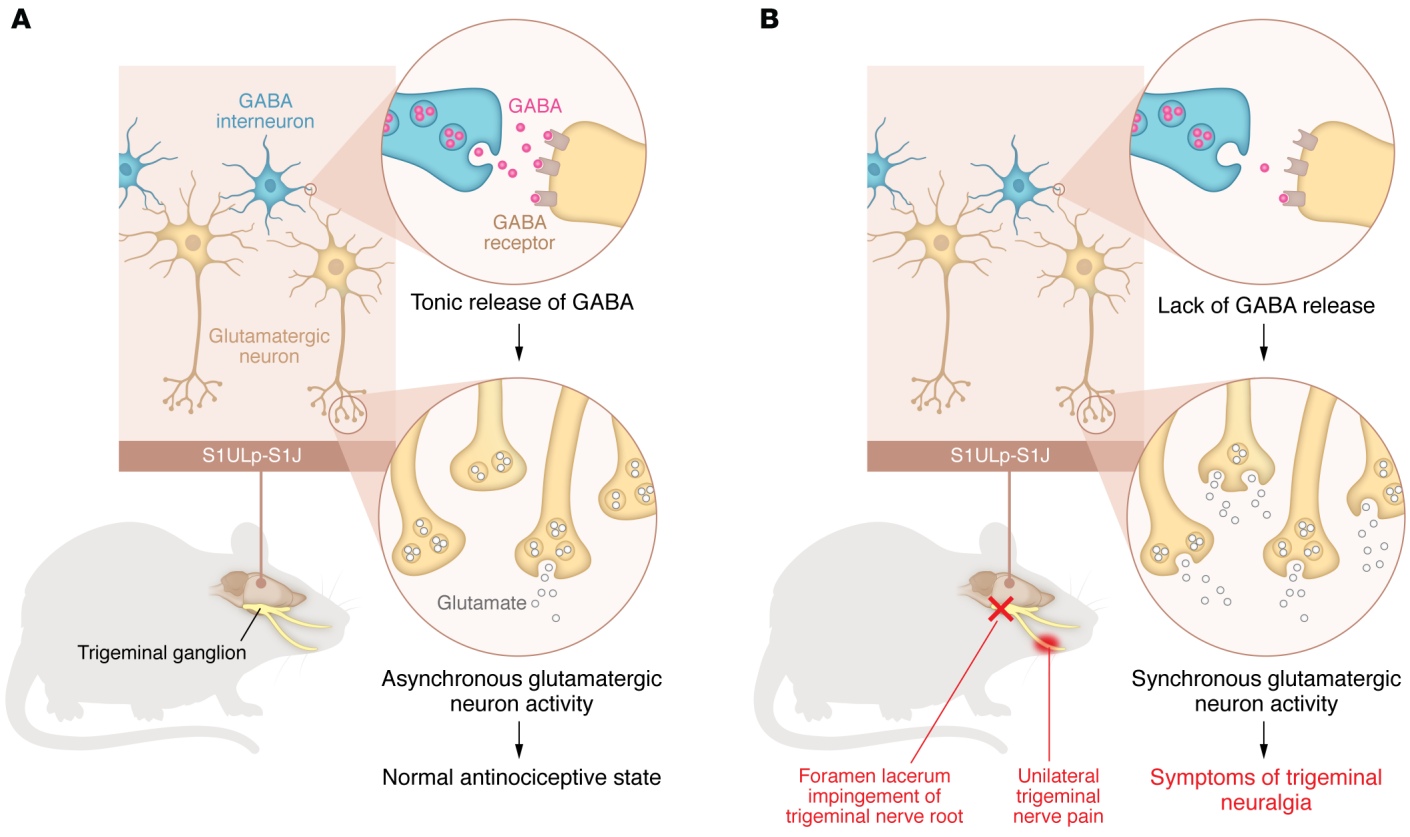
Coordination of glutamatergic activity in the somatosensory cortex drives neuropathic pain phenotypes. (**A**) Somatosensory afferent information from the face is transmitted from the trigeminal ganglion via the trigeminothalamic tract to S1. In the absence of nociceptor activation in the territory covered by the trigeminal ganglion, GABA, which is tonically released by interneurons, binds to GABA receptors on glutamatergic neurons in the S1ULp–S1J region, which produce asynchronous glutamatergic neuron activity. (**B**) Impingement of the trigeminal nerve root at the foramen lacerum by surgically introducing a gelatin sponge in mice replicates symptoms of human TN. A lack of GABA secretion via GABAergic interneurons releases the functional inhibition of glutamatergic neurons, increasing glutamate within the extracellular space and contributing to an excitatory response. Synchronous glutamatergic neuron activity in the S1ULp–S1J region produces symptoms of TN.

Even more important, Ding, Fischer, and colleagues (19) then used the model to make important observations regarding cortical dynamics that underlie the mechanism of TN pain (Figure 1). Most studies of central sensitization focus on changes in the periphery at the level of the nociceptor and its synaptic connection at the dorsal horn of the spinal cord. This study used state-of-the-art techniques, including two-photon in vivo calcium imaging in awake mice and chemogenetic manipulation of targeted recombination in active populations (TRAPPed) neural circuits to show that unrestrained, synchronous glutamatergic activity in a specific region of the somatosensory cortex — the primary somatosensory cortex upper lip and jaw (S1ULp–S1J) — drove the pain phenotypes. The authors further demonstrated that this unrestrained activity was due to hypoactive GABAergic interneuron activity. Important control experiments showed that manipulations of an adjacent somatosensory cortex region and masseter muscle atrophy were not mechanistically linked to TN-like pain behaviors (19).

But the study by Ding, Fischer, and co-authors (19) also brings up questions for further investigation. One of the most intriguing questions in pain research relates to the point at which acute, temporary sensitization of the nociceptive system becomes persistent and the mechanism driving this persistence. It wasn’t long ago that the concept of chronic pain changed from using time as a reference for its establishment to considering the development of plastic changes in the nociceptive system as the driving element for pain persistence. Ding, Fischer, and colleagues provide important information regarding the interaction between peripheral sensory neurons and specific central circuits in this process. The authors found that the activity from peripheral sensory neurons (the nociceptive response to trigeminal root compression) triggered specific localized cortical reflexes (an increase in glutamatergic and a corresponding decrease in GABAergic activity in the S1ULp–S1J area) that, in a coordinated manner, were necessary to produce the pain phenotype (Figure 1). Interestingly, this interaction only lasted as long as the lesion was present, which can be interpreted as a lack of involvement of central neuroplasticity in FLIT-induced pain. Although the seven-day period between the induction of TN and the beginning of the evaluation of pain was adequate to determine the presence of painful neuropathy, the magnitude of information coming from the periphery might not have been intense enough to stimulate plasticity mechanisms, since the removal of the cause (decompression of the trigeminal root) alleviated the symptomatology. As one of the challenges in neuropathic pain is its persistence even after the cause is removed, the authors’ observation might be an indication that the development of chronic pain depends on a prolonged stimulation of specific areas in the brain (S1ULp–S1J, for example) by peripheral input, eventually producing neuroplastic changes that lead to independent activity. Thus, one can question whether the phenotype produced by the FLIT procedure meets the criteria for chronic pain, since the current definition considers the key role of neuroplasticity in pain maintenance. While the authors have not addressed this point in their study, the proposed model and methodology provide an excellent tool to systematically investigate the interaction between the peripheral and central nervous systems that leads to the establishment of chronic pain and the critical time point at which chronic pain occurs (19).

## Future opportunities

Along with efforts to produce better preclinical models, progress is being made in other areas of chronic pain research. There is an increased emphasis on nonpharmacologic interventions that focus on the biopsychosocial components of pain (22). Many investigators are seeking to identify biochemical (23) and phenotypic (24) biomarkers to guide a precision medicine approach targeting the right treatment at the right dose to the right patient at the right time. Ultimately, if we hope to achieve greater success in discovering effective treatments for chronic pain compared with those of the past six decades, it will depend on an entire disruption of the drug discovery strategies of the past. Instead of identifying potential therapeutic targets in animal models, pain target identification must be made using information from human cells and pain networks (25). Testing of potential drug candidates must then be carried out in animal models that possess greater face, construct, and predictive validities, such as those offered by the FLIT model. Finally, clinical trials should then be designed that will utilize biomarker-selected patient populations. At present, we lack enough knowledge in all three of these areas to successfully implement this strategy. But as the study by Ding, Fischer, and colleagues (19) demonstrates, progress is being made toward more effective treatments for chronic pain.

## Version 1 03/01/2023

Electronic publication
